# On the Use of Ridge Gap Waveguide Technology for the Design of Transverse Stub Resonant Antenna Arrays

**DOI:** 10.3390/s21196590

**Published:** 2021-10-02

**Authors:** Javier Benavides-Vazquez, Jose-Luis Vazquez-Roy, Eva Rajo-Iglesias

**Affiliations:** 1 Indra Sistemas, S.A., 28850 Torrejon de Ardoz, Spain; 2 Signal Theory and Communications Department, Universidad Carlos III de Madrid, 28911 Leganes, Spain; jvazquez@tsc.uc3m.es (J.-L.V.-R.); eva@tsc.uc3m.es (E.R.-I.)

**Keywords:** continuous transverse stub (CTS), ridge gag waveguide (RGW), ka-band, resonant antenna

## Abstract

This paper presents some considerations on the design of a novel antenna consisting of the combination of a transverse stubs (TS) array excited by Ridge Gap Waveguides (RGWs), as well as a discussion of the experimental results obtained from a prototype that was manufactured and measured. A combination of Continuous Transverse Stubs (CTSs) is used as the starting point. Subsequently, the CTSs are modified to include some metallic blockers that split each CTS into a combination (array) of shorter TSs. This is performed in order to excite each individual TS column using a different RGW; thus, ensuring a close to uniform field distribution in the transverse plane of the TS arrays. Hence, the directivity of the antenna is increased. As a series-feed configuration is considered, the antenna keeps a resonant behaviour, having a narrow-band response. A Corporate Feeding Network (CFN) using the aforementioned RGW technology placed in the same layer as the rest of the antenna is included in the design. The radiating area of the antenna is, finally, $5.88\lambda_{0}\times 7.12\lambda_{0}$ with a simulated peak gain of 26.2 dBi and a Side Lobe Level (SLL) below −13 dB. A prototype is manufactured and tested. The simulated and measured radiation patterns maintain similar shapes to those of the simulations, with very similar angular widths in both main planes, although the frequency corresponding to the highest directivity changes to 31.8 GHz. A matching bandwidth of 517 MHz and a gain of 24.5 is, finally, achieved at that frequency.

## 1. Introduction

Nowadays, with the trend of moving up in frequency communication services, it is common to see antennas working at very high-frequency bands. Moreover, antenna integration has become a key issue for the radio-frequency community.

Some applications, such as on-the-move SATCOM communications, are following the aforementioned trends, besides many others. Consequently, very compact designs are needed in order to integrate these antennas above different structures (aircraft, ground vehicles, etc.). As for any other satellite application, the gain of an antenna is a key performance indicator.

In this context, Continuous Transverse Stub (CTS) arrays appear as interesting candidates for the antenna designer. With very high directivity values, compact designs and high aperture efficiencies, these elements have demonstrated several benefits over other antenna designs (e.g., slotted waveguides or microstrip patches) [1,2,3].

In their series-feed resonant form, i.e., terminating the antenna with a short circuit [4], narrowband responses are expected. For those cases in which a true-time delay implementation [5] is selected, e.g., [6], wider bandwidths can be achieved at the cost of increasing the design and manufacturing complexity by using a parallel feed strategy.

**Figure 1:**
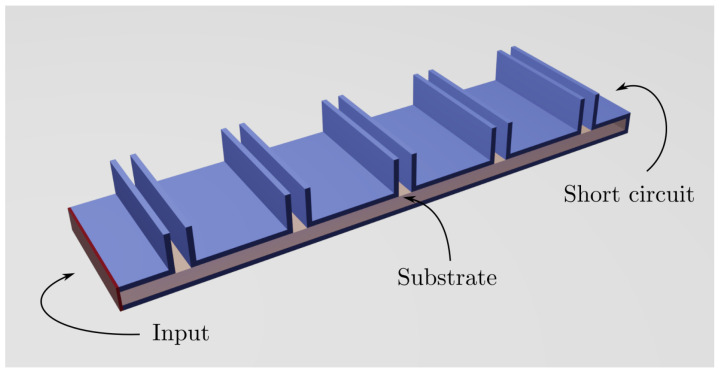
Array of open-circuited CTSs in resonant configuration.

Considering a resonant design where the radiating elements are located in the positions of the maxima of a standing wave, a $\lambda_{g}$ spacing between consecutive CTS radiating elements is needed. For this reason, a low-loss dielectric is used in the host waveguide to avoid grating lobes in the resulting radiation pattern by making $\lambda_{g}<\lambda_{0}$. On the contrary, air-filled stubs are used in our proposal in the radiating part of the antenna. Figure 1 depicts this situation.

When radiating CTSs are used in a Parallel-Plate Waveguide (PPW) structure, a uniform E-field distribution is only achieved inside the stubs when Perfect Magnetic Conductor (PMC) boundary conditions are used in their lateral ends. Any other field distribution will result in a loss of directivity. In our case, PEC boundary conditions are used laterally to enclose the full structure so the fundamental mode for the stubs is the $TE_{10}$, but due to the long width of the elements, many higher order modes can be present depending on the excitation mechanism used.

**Figure 2:**
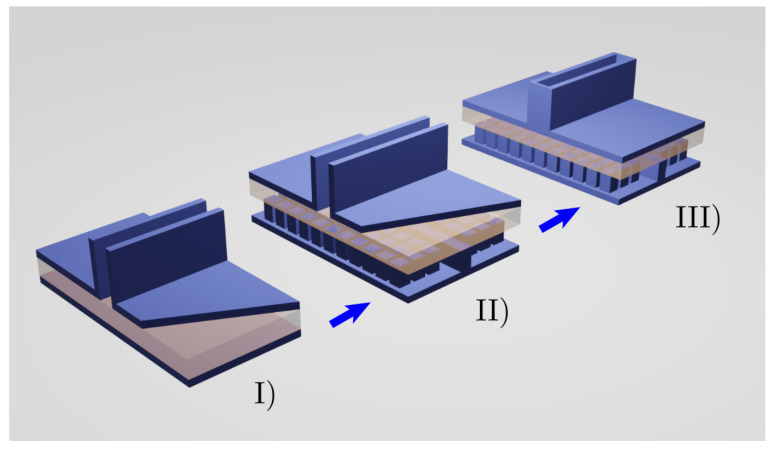
Basic CTS in a PPW (I), a CTS excited by an RGW (II) and a laterally-limited CTS excited by an RGW (III).

RGW technology [7,8,9], with the so-called Bed of Nails (BoN) [10], can readily be employed to excite slot antennas [11,12,13] and this approach can also be used to excite a CTS, as shown in Figure 2, that shows the conventional CTS implementation in a PPW (I), the same laterally infinite structure but excited by a centred RGW (II), and, finally, the RGW exciting a CTS (III) which is laterally limited by a PEC BC imposed by two metallic blockers.

**Figure 3:**
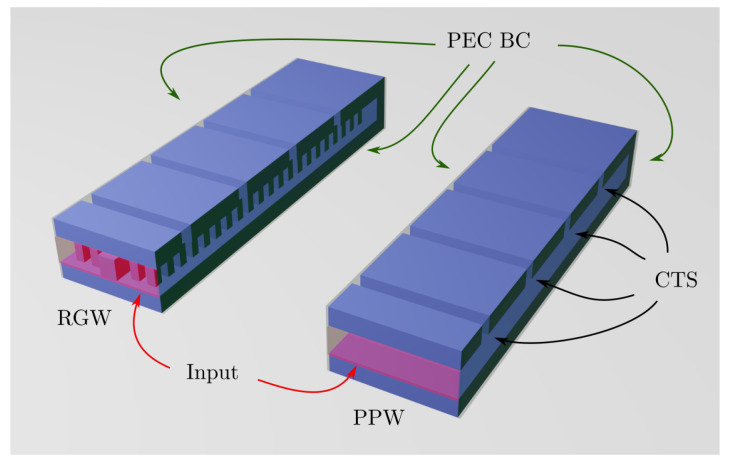
Comparison between a single ridge exciting a column of CTSs (**left**-hand figure) and a PPW design (**right**-hand figure), both with lateral PEC boundary conditions.

In order to prove that exciting an array (column) of CTSs using RGW increases the directivity in comparison with a standard PPW implementation, some simple tests are carried out using simulation schemes such as the one shown in Figure 3. The models contain linear arrays of TSs, which are laterally terminated with PEC boundary conditions. Note that the stubs are now made as deep grooves in a solid sheet metal. The directivity is found to be higher in the RGW case compared to the PPW case due to the field distribution achieved in the stubs. The lateral extent of the stubs is of the order of the wavelength.

**Figure 4:**
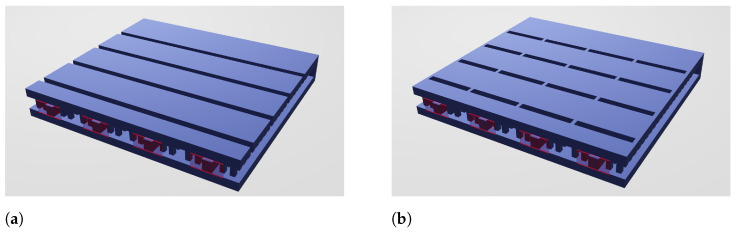
CTS excitation by means of a combination of RGW: conventional CTSs (**a**) and short TSs (**b**).

In a first attempt, and with the idea of achieving a field distribution in the stubs as uniform as possible, equally spaced RGWs fed with the same amplitude are used to excite CTSs arranged in a linear array in the way showed in Figure 4a. The aim is also to achieve a distribution as symmetrical as possible. The simulation results show a not so clean field distribution in the cross section due to the uncontrolled excitation of higher order modes, so it was decided to split each individual CTS into smaller TSs by means of metallic blockers such as the ones described in Figure 2 (III). Hence, the resulting antenna could be assimilated to an N×N TS array (see Figure 4b).

As a result, in [14], a proof of concept is designed and simulated in order to check the potential of an 8×8 antenna array at 31 GHz, integrating RGW technology with TS radiating elements. A 1:8 power splitter implemented in air-gap RGW technology is also designed to properly excite the eight ridges that feed the TS elements. Given that the obtained results are promising, a prototype is manufactured and measured. The results of those previous simulations and the subsequent measurements are discussed next, as well as the convenience of using solutions based on the aforementioned combination of elements.

## 2. Antenna Design

### 2.1. Bed of Nails (BoN)

The Bed of Nails (BoN),composed of rectangular pins, was employed in order to guarantee that no field could propagate outside the path provided by the ridges, ensuring, hence, a quasi-uniform field distribution along the modified CTS radiating elements.

This structure was based on the principle that no modes can propagate in between a Perfect Magnetic Conductor (PMC) in parallel with a Perfect Electric Conductor (PEC) [15], provided that the height separating both plates, $h_{g}$, is less than $\lambda_{g}/4$ [9]. Moreover, it was required to have a pin height, $h_{p}$, of around $\lambda_{0}/4$, in order to ensure the PMC condition at the top of the pin while having a PEC boundary sustaining the BoN, and a small pin period as compared to the free space wavelength.

Two different propagating sections were considered: the first regarding the CFN, in which the quasi-TEM mode in between the upper surface of the ridge and the upper metallic plate was going to propagate through air, and the second regarding the radiating part of the structure, in which the substrate RT/duroid™ 5880LZ ($\epsilon_{r}=2$, tanδ=0.0027) was employed.

By simulating a unit cell comprising both the pin itself and the dielectric gap above it, and setting a periodic boundary in all directions except in the upper and lower parts of the pin which were set to be electric boundaries, it was possible to obtain the dispersion diagram; hence, checking that no modes were going to propagate at the operating frequencies. This analysis had to be conducted both considering air and RT/duroid™ 5880LZ as the dielectrics placed above the pin. Table 1 shows the resulting geometry for the design of the BoN.

**Figure 5:**
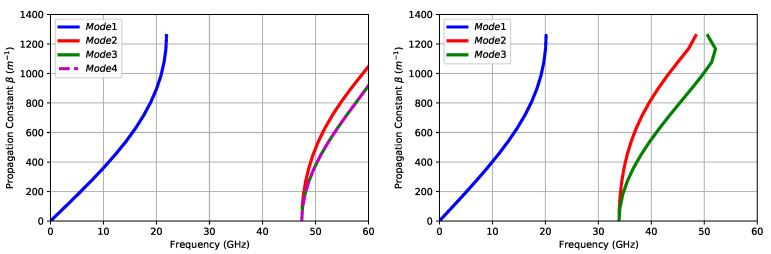
Dispersion diagrams of the unit cell pin configuration for air (**left**) and RT/duroid™ 5880LZ (**right**).

As it can be observed in Figure 5, neither when using the 5880LZ substrate, nor with air, did the modes propagate at the desired operating frequencies.

### 2.2. Corporate Feeding Network

**Figure 6:**
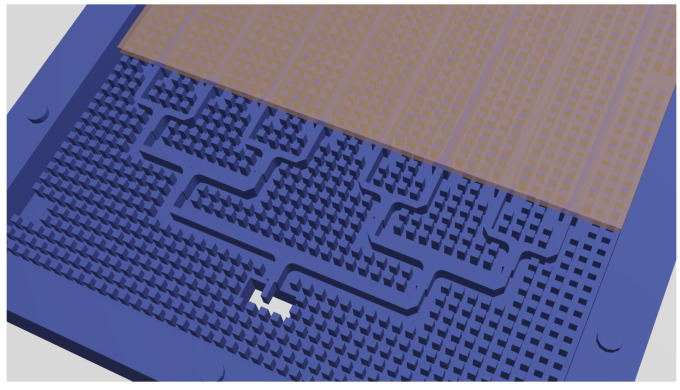
CFN implemented with air gap RGW (note the substrate used in the slow-wave RGW that feeds the TS array).

In order to feed the eight ridges which excite the TS columns, a CFN of size 1×8 was employed (see Figure 6). This CFN made use of RGW + BoN technology and employed air as the dielectric confining the propagating field along the ridges. This was preferred in terms of ohmic losses rather than using a different dielectric.

**Figure 7:**
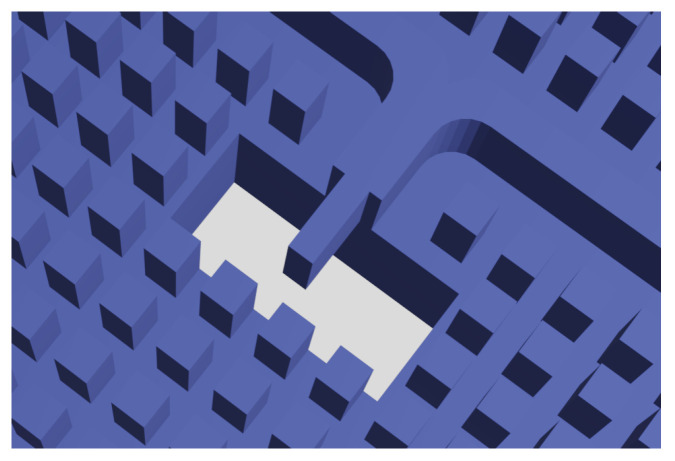
WR28 to RGW transition.

Firstly, a WR28 (standardized waveguide which can operate at the centre frequency of 31 GHz) to ridge gap waveguide transition was designed after [16]. This design is shown in Figure 7.

Regarding the CFN design, λ/4 sections were used to match the input of every −3 dB power splitter in the corporate network. This type of element was also used at the outputs of the CFN, since the impedance seen at the input of the RGW of the radiating part of the structure had to be compensated due to the use of different dielectrics at each side of the discontinuity.

**Figure 8:**
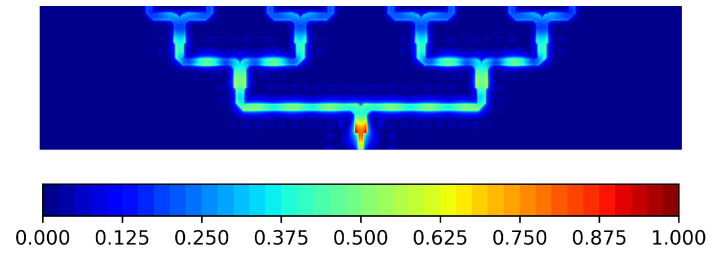
Magnitude of the normalized $E_{z}$ at $f_{0}=31$ GHz shown in the air gap of the CFN.

The CFN distributes the power equally at each arm split. This behaviour can be clearly seen in Figure 8, where the magnitude of the vertical component of the electric field is displayed.

**Figure 9:**
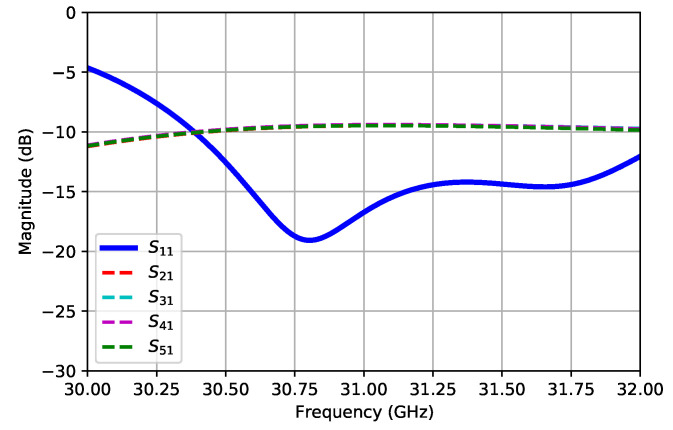
Simulated S-parameters of the corporate feeding network.

For the purpose of simulating the CFN, CST Studio Suite^®^ with its Time Domain Solver was employed [17]. Figure 9 shows both the reflection and transmission coefficients for the corporate feeding network. This structure included the WR28-to-ridge transition, which was, indeed, the feeding port (number one). As the structure was symmetric, only the transmission to the right-hand side output ports (two to five) was considered. A good impedance matching at the input port was observed for the frequency of operation (in fact, this structure exhibited a wideband response, as expected), as well as a good transmission to all output ports with an Insertion Loss of around 1 dB.

### 2.3. Radiating Structure

**Figure 10:**
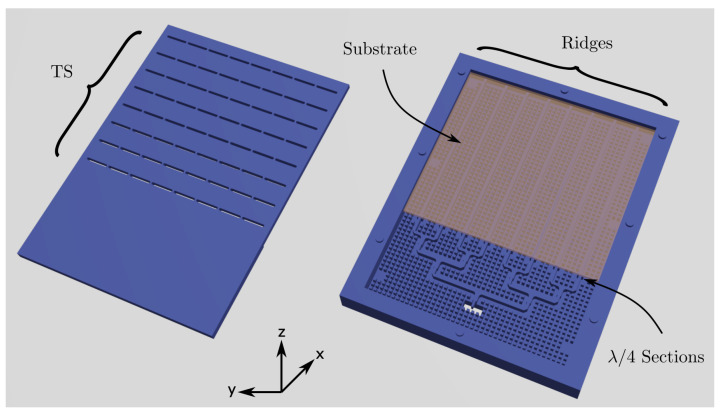
Schematic of the proposed antenna design.

The schematic of the antenna design can be found in Figure 10, while all geometric parameters related with the antenna are summarized in Table 2.

**Figure 11:**
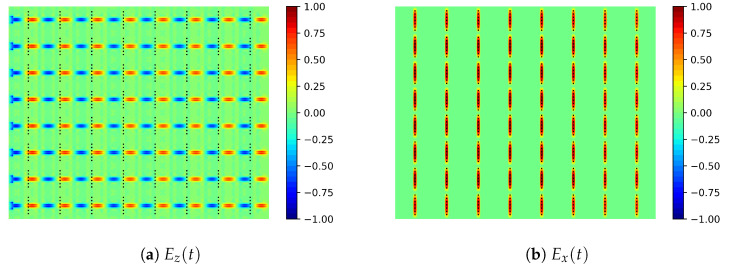
Simulated normalized $E_{z}\left(t\right)$ at the ridge level in the radiating section (ridges fed from the left side) and $E_{x}\left(t\right)$ at the aperture level at 31 GHz.

Figure 11 shows the instantaneous electric field components $E_{z}$ and $E_{x}$ on cutting planes at the ridges and the aperture level. Although Figure 11a shows a slight phase shift from left to right in the excitation of the TS, Figure 11b shows a field maxima in practically all the radiating elements; therefore, enabling broadside radiation.

**Figure 12:**
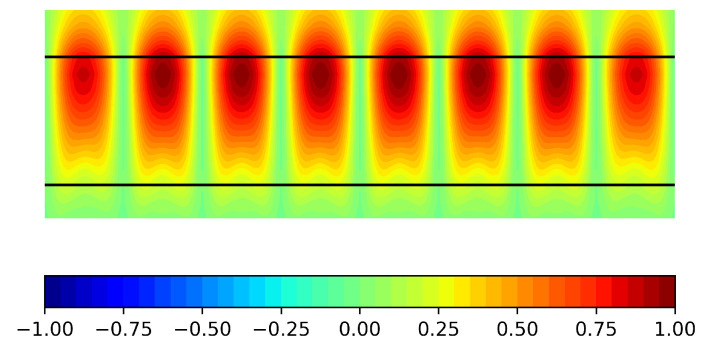
Simulated normalized $E_{x}\left(t\right)$ field component at the cross section for the first row at 31 GHz (TS vertical limits indicated in black; y and z dimensions not to scale).

Regarding the transverse plane, Figure 12 displays the instantaneous $E_{x}$ field distribution inside the first TS row at the nominal frequency. It can be seen how the field distribution fills much of the aperture. This would obviously contribute to the increase in the antenna directivity.

**Figure 13:**
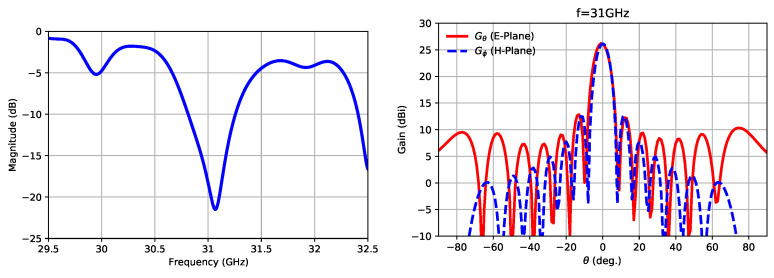
Simulated $S_{11}$ and gain radiation patterns for the antenna at 31 GHz.

Finally, Figure 13 includes the simulation response of the designed antenna in input matching and radiation patterns. A maximum gain of 26.2 dBi was expected at 31 GHz.

## 3. Experimental Results and Discussion

**Figure 14:**
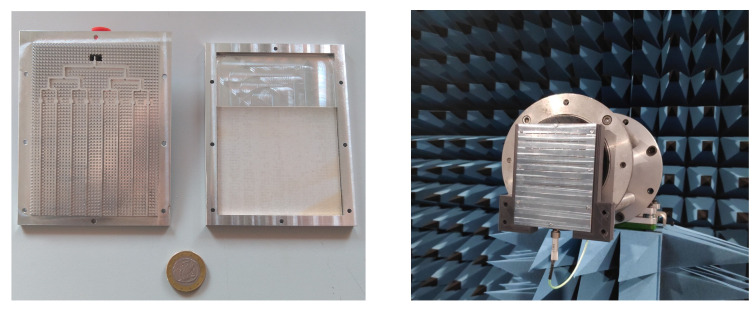
Manufactured prototype: excitation network and substrate arrangement (**left**) and assembled antenna in anechoic chamber showing the radiating side (**right**).

Figure 14 shows a picture of the manufactured prototype, where the excitation network and the substrate arrangement can be identified, as well as the assembled antenna in the anechoic chamber showing the radiating side with the TS array. The antenna was manufactured using conventional milling techniques.

**Figure 15:**
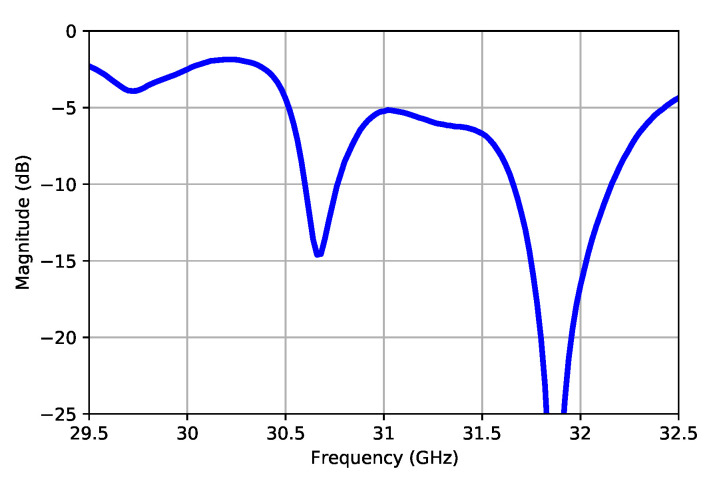
Measured $S_{11}$ for the manufactured antenna.

**Figure 16:**
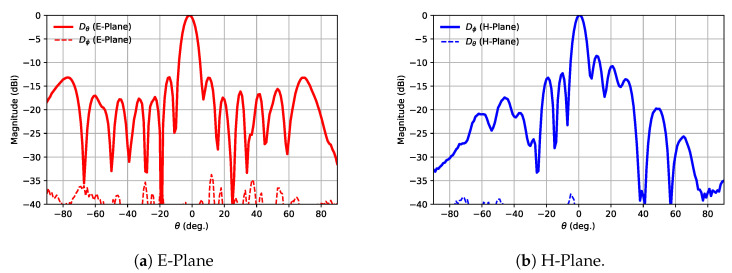
Measured normalized radiation pattern at 31.8 GHz.

After measuring the antenna, it was found that, the desired radiation response had shifted in a matched bandwidth towards the high frequencies (Figure 15) and the best radiation results were obtained at 31.8 GHz (Figure 16), which corresponded to a 2.7% shift from the target frequency. It was also observed how the E-field pattern was tilted $-2^{\circ }$, approximately.

Based on the results obtained, a study of the effect of the substrate permittivity on the antenna response was carried out. For this purpose, different simulations were performed for ±2% variations in that value. These variations may be related not only to the manufacturing tolerances of the substrate, but also to the presence of small air gaps due to the lack of flatness of the substrate itself and the metal parts that make up the antenna.

**Figure 17:**
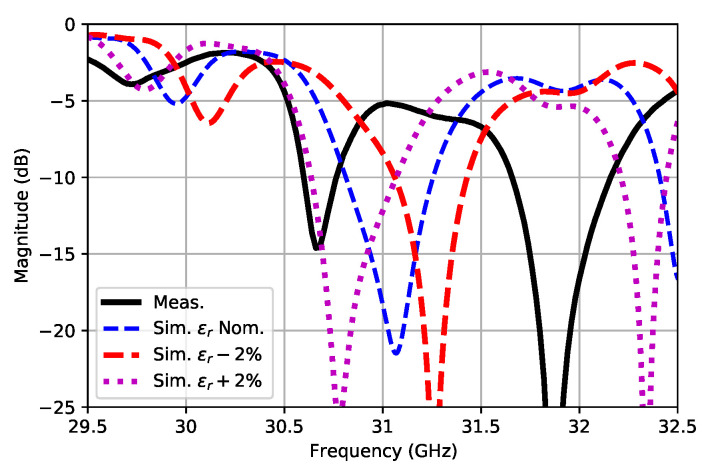
Measured vs. simulated $S_{11}$ for different permittivities.

Figure 17 shows the simulated $S_{11}$ for the nominal permittivity and for ±2% variations. Clearly, the effective permittivity was lower than initially assumed for the ideal substrate. As we were using the dielectric as a form of a slow-wave implementation, its exact value when manufacturing the prototype was critical. The distance in between adjacent maxima positions of the standing wave strongly depended on this value, so if it changed with respect to the selected one when designing the antenna, the radiating stubs would not be precisely placed at the top of each standing wave maximum; thus, affecting the impedance matching of the antenna and, also, altering the radiation patterns with some possible main lobe tilting and a side lobe appearance.

**Figure 18:**
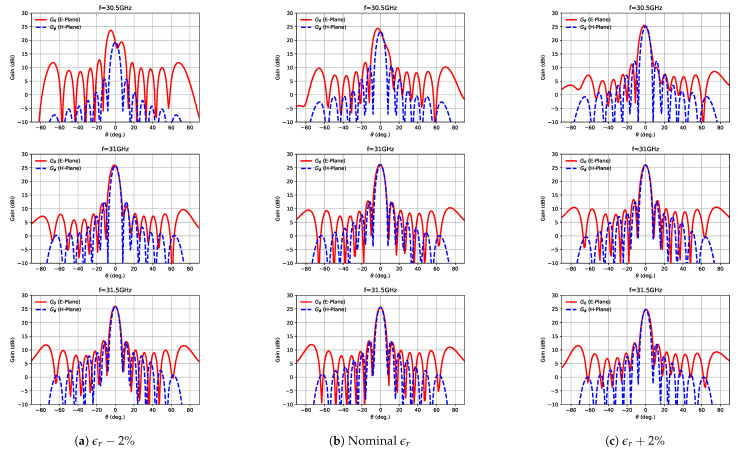
Simulated radiation patterns at 30.5, 31 and 31.5 GHz for different substrate permittivities.

**Figure 19:**
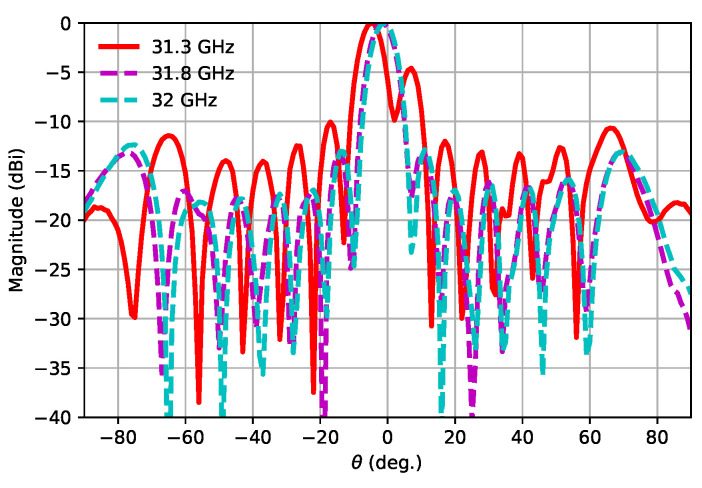
Measured E-plane radiation patterns ($E_{\theta }$) for different frequencies.

Figure 18 represents the simulated E-plane and H-plane radiation patterns for different permittivities at 30.5, 31 and 31.5 GHz. As can be seen, due to the ±2% variation in the relative permittivity, some differences were observed in the radiation patterns. The strongest variations were produced for the lowest analysed frequency, i.e., 30.5 GHz, with some tilting observed and the appearance of a secondary lobe near the main one. Figure 19 represents the E-plane $E_{\theta }$ radiation patterns measured at three different frequencies. It can be seen how those patterns corresponded approximately to those represented in Figure 18 (left column), relating again to a permittivity lower than the nominal one.

In Table 3, all simulated and measured results are shown and compared. Table 4 shows a comparison with other similar antennas employing CTS technology, in particular with a true-time delay implementation.

## 4. Conclusions and Future Work

It was demonstrated how the integration between RGW technology and radiating TSs made it possible to obtain a close to uniform field distribution at each row in the array. This behaviour had a direct impact on the maximization of the antenna gain, which resulted in a peak value of 24.5 dBi in the measurements. Moreover, pencil-beam broadside radiation patterns were obtained in both principal planes, with a maximum angular width of around $8^{\circ }$ for the worst case.

Good results were obtained both at simulation and measurement levels, both in impedance matching and radiation characteristics. Specifically, measurements showed an absolute matching bandwidth of 517 MHz. On the other hand, the best radiation results were obtained at 31.8 GHz. Besides this 2.5% frequency shift, the E-plane radiation pattern showed a small main lobe tilt. The H-plane measured radiation pattern agreed well with simulations in its main lobe. A small increment in the SLL was observed in both principal planes.

RGW technology allows to implement the CFN in the same layer as for the TS array, which produced a direct simplification for the manufacturing process. This fact also made the antenna very compact.

This prototype could be suitable for narrowband applications requiring flat and compact antenna arrays with a high gain if the described problematic effects could be mitigated, namely:

- The exact value of the substrate’s relative permittivity plays a critical role on the antenna performance, given that its resonant response is directly affected by this figure. As per commented before, when this value changes, the standing wave maxima positions move. This fact has a direct impact on the impedance bandwidth as well as on the E-plane radiation patterns, both in terms of the main lobe tilt and gain.
- There is a trade-off between the enhancement of the antenna directivity, achieved thanks to the good illumination obtained at the antenna aperture, and the insertion losses produced at the CFN that are used to feed the array. The evaluation of this trade-off is part of the work being conducted in ongoing research.

## Figures and Tables

**Table 1:** BoN geometry.

Parameter	Parameter Description	Value (mm)
$h_{p}$	Pin height	2.42
*s*	Pin side width	1
*p*	Pin period	1.75
$w_{s}$	Separation between a ridge and its adjacent pin column	1.75
$h_{g}$	Height of the substrate gap	1.27
$h_{air}$	Height of the air gap	0.5

**Table 2:** Antenna geometry.

Parameter	Parameter Description	Value (mm)
$w_{trans}$	Ridge width of the WR28 transition	0.75
$w_{imp}$	$\lambda_{0}/4$ ridge impedance transformers width	1.5
$w_{corp}$	Corporate feeding ridges width	1
$h_{air}$	Air gap at the feeding network	0.5
$h_{g}$	Substrate height at the radiating section	1.27
$l_{s}$	TS length	2
$w_{s}$	TS width	0.9
$d_{r}$	Distance between ridges	8.75
$d_{s}$	Distance between TS	8
$l_{i}$	Matching section length (dielectric discontinuity)	1.69
$w_{i}$	Matching section width (dielectric discontinuity)	2.25

**Table 3:** Comparison between simulation and measurement results.

Parameter	Parameter Description	Simulation 31 GHz	Measurement 31.8 GHz
*B*	Matching bandwidth (RL > 10 dB)	446 MHz	517 MHz
*G*	Antenna gain	26.2 dB	24.5 dB
$\Delta \theta_{-{3\mathrm{dB}}_{E}}$	E-plane angular width (−3 dB)	7.8${}^{\circ }$	$8^{\circ }$
$\Delta \theta_{-{3\mathrm{dB}}_{H}}$	H-plane angular width (−3 dB)	7${}^{\circ }$	$6.5^{\circ }$
$SLL_{E}$	E-plane SLL	−13.2 dB	−13.1 dB
$SLL_{H}$	H-plane SLL	−13.8 dB	−8.7 dB

**Table 4:** Comparison with other similar state of the art antennas.

Reference	Electrical Size ($\lambda_{0}^{2}$)	Percentage Bandwidth (%)	Gain (dBi)
[18]	12.3×6.7	42.4	>26.8
[19]	26.1×16.9	12	28.9
[20]	43.2×25.1	19	30.8
This work	5.88×7.12	1.63	24.5

## Abbreviations

The following abbreviations are used in this manuscript:

BC	Boundary Condition
BoN	Bed of Nails
CFN	Corporate Feeding Network
CTS	Continuous Transverse Stub
PEC	Perfect Electric Conductor
PMC	Perfect Magnetic Conductor
PPW	Parallel-Plate Waveguide
RGW	Ridge Gap Waveguide
SLL	Side Lobe Level
TEM	Transverse Electro-Magnetic
TS	Transverse Stub
